# 2021 European Society of Cardiology guidelines on cardiac pacing and cardiac resynchronisation therapy: Statement of endorsement by the NVVC

**DOI:** 10.1007/s12471-024-01927-y

**Published:** 2025-01-21

**Authors:** Alexander H. Maass, Anton Tuinenburg, Gideon Mairuhu, Miriam C. Faes, Theo J. Klinkenberg, Sanne Ruigrok, Marjolein Koster, Bernardine H. Stegeman, Justin G. L. M. Luermans

**Affiliations:** 1 Department of Cardiology, University Medical Centre Groningen, University of Groningen, Groningen, The Netherlands; 2 Department of Cardiology, University Medical Centre Utrecht, Utrecht, The Netherlands; 3 Flevo Hospital, Almere, The Netherlands; 4 Amphia Hospital, Breda, The Netherlands; 5 Department of Cardiothoracic Surgery, University Medical Centre Groningen, University of Groningen, Groningen, The Netherlands; 6 Harteraad, Den Haag, The Netherlands; 7 Department of Cardiology, Amsterdam University Medical Centre, Amsterdam, The Netherlands; 8 Knowledge Institute of the Dutch Association of Medical Specialists, Utrecht, The Netherlands; 9 Department of Cardiology, Cardiovascular Research Institute Maastricht, Maastricht University Medical Centre, Maastricht, The Netherlands

**Keywords:** Pacemaker, Conduction system, Shared decision making, Indication

## Abstract

The European Society of Cardiology (ESC) has updated its guidelines on cardiac pacing and cardiac resynchronisation. As the majority are class II recommendations (61%) and based on expert opinion (59%), a critical appraisal for the Dutch situation was warranted. A working group has been established, consisting of specialists in cardiology, cardiothoracic surgery, geriatrics, allied professionals in cardiac pacing, and patient organisations with support from the Knowledge Institute of the Dutch Association of Medical Specialists. They assessed the evidence leading to the recommendations and the suitability for daily Dutch practice. Several recommendations have been amended or omitted altogether if a conflicting Dutch guideline has recently been published, such as a guideline on performing magnetic resonance imaging in patients with cardiac implantable electronic devices. The recent Dutch guideline on implantable cardioverter defibrillator implantation in patients with non-ischaemic cardiomyopathy has recommended implanting cardiac resynchronisation therapy devices without a defibrillator function. Shared decision making has received a more prominent role in the ESC guidelines and is discussed in more detail in this document. The recommendations given in this document are intended for health care professionals involved in the care of patients with an indication for cardiac pacing and are approved by the participating professional societies and the patient organisation Harteraad.

## Introduction

Cardiac pacing can improve patients’ quality of life and in certain conditions can also improve outcome. Whereas conventional pacing was established decades ago, novel developments such as cardiac resynchronisation therapy (CRT) and, more recently, leadless pacing and conduction system pacing (CSP) require international guidelines to be updated regularly. With regard to the situation in Europe, an update on the European Society of Cardiology (ESC) guidelines for cardiac pacing and CRT was published in 2021 [1] to replace the 2013 guidelines [2].

Few randomised controlled trials (RCTs) have been performed on cardiac pacing. Therefore, many recommendations have a level of evidence and also a level of recommendation that are lower than in other guidelines. In fact, only 39% of recommendations are class I or III and only 11% have a level of evidence A (summarised in Tab. 1).

**Table 1:** Number of recommendations per class of recommendation and level of evidence

Class of recommendation/Level of evidence	Number	Percentage of total
I/A	9	7.5%
I/B	6	5%
I/C	19	16%
IIa/A	2	2%
IIa/B	18	15%
IIa/C	28	23.5%
IIb/A	0	0%
IIb/B	6	5%
IIb/C	18	15%
III/A	2	2%
III/B	5	4%
III/C	5	4%

As in other guidelines, there are quite a number of gaps in the evidence. In particular, CSP has been introduced in daily clinical practice with little scientific evidence.

With this amendment to the current ESC guidelines, we aim to achieve several goals. First of all, recommendations with weaker evidence are reflected on according to daily Dutch clinical practice. Secondly, gaps in the evidence are addressed in addition to a reflection on CSP. Last but not least, shared decision making (SDM) has been underappreciated in earlier guidelines, especially considering that many decisions in cardiac pacing are not made to improve patient outcome but to improve quality of life. This article summarises recent recommendations regarding SDM, as well as the officially published addendum.

## Methods

At the initiative of the Dutch Society for Cardiology (NVVC), an addendum to the ESC guideline ‘Cardiac pacing and cardiac resynchronisation therapy’ was developed. The development of the addendum was supported by the Knowledge Institute of the Dutch Association of Medical Specialists (Knowledge Institute) and was financed by the Foundation for Quality Funds for Medical Specialists. The financier has had no influence whatsoever on the content of the addendum.

This addendum is written for all members of the professional groups of cardiologists, cardiothoracic surgeons and clinical geriatricians who are involved in the care of patients with an indication for cardiac pacing. In addition, this guideline is intended to inform healthcare providers who are otherwise involved with these patients.

### Composition of working group

To develop the addendum, a multidisciplinary working group was established in 2023, consisting of representatives of all relevant specialties involved in the care of patients with an indication for cardiac pacing.

### Declarations of interest

The Royal Dutch Medical Association code to prevent undue influence due to conflicts of interest has been followed. All working group members have stated in writing whether they have had direct financial interests (employment with a commercial company, personal financial interests, research funding) or indirect interests (personal relationships, reputation management, knowledge valorisation) in the last 3 years. An independent committee of the Knowledge Institute evaluated the reported potential conflicts of interest. The signed declarations of interest can be requested from the Knowledge Institute.

### Input on patient perspective

Attention was paid to the patient perspective by conducting interviews with patients and the participation of a representative patient association in the working group (see Summary of patient perspective). Harteraad conducted a literature search, held in-depth interviews with six patients and analysed contributions in a closed community for patients with cardiac arrhythmia. The draft addendum was also submitted to Harteraad for comment and any comments submitted have been reviewed and processed.

### Method

#### Drafting of addendum

The working group assessed the recommendations for applicability in the Netherlands. Possible adjustments were discussed during several meetings.

#### Comment and authorisation phase

The draft addendum was submitted to the relevant (scientific) associations and Harteraad for comments. The comments were collected and discussed with the working group. In response to the comments, the draft addendum was adjusted and finally adopted by the working group. The final addendum was submitted to the participating scientific associations and Harteraad for authorisation and authorised or approved by them.

## Amended recommendations

Most of the recommendations were not amended and were endorsed by the working group.

Tab. 2 shows a brief overview of the changes/additions to and clarifications of some amended recommendations of the ESC guidelines for cardiac pacing and CRT. The following text provides details and justifications for any changes made.

**Table 2:** Changes and additions to/clarifications of the recommendations in the European Society of Cardiology (ESC) guidelines

ESC guideline	Dutch cardiac pacing working group recommendation
Chapter 6.3 ‘Cardiac resynchronisation therapy in patients with persistent or permanent atrial fibrillation’ (2) In patients with symptomatic AF and an uncontrolled heart rate who are candidates for AVJ ablation (irrespective of QRS duration): CRT may be considered in patients with HFpEF	*The following was added to the recommendation:* Placing a CRT device involves complications (the risk of complications is higher than with a 1-chamber or 2‑chamber device). It is important to make the decision regarding the installation of a CRT device through shared decision making. In patients with permanent AF of > 6 months duration and earlier heart failure hospitalisation, CRT in combination with AV node ablation should be considered instead of medical therapy
Chapter 6.6 ‘Benefit of adding implantable cardioverter defibrillator in patients with indication for cardiac resynchronisation therapy’ In patients who are candidates for CRT, implantation of a CRT‑D should be considered after individual risk assessment and using shared decision-making	*The following was added to the recommendation*: It is recommended that the Dutch Indication Guideline for Primary Prevention ICD Placement in patients with non-ischaemic cardiomyopathy be followed
Chapter 7.2 ‘His bundle pacing’	*The following two recommendations were added*: A proposed treatment with CSP versus conventional RV or CRT pacing should be discussed with the patient, weighing the advantages and disadvantages, including potential other short- and long-term complications (see ‘Table 5: Advantages and limitations of HBP and of LBBAP’ in the EHRA consensus document [10]). The final decision should be made in consultation with the patient. The advice on the practical application of CSP as stated in the ‘Table of advice’ chapter in the EHRA consensus document [10] has been adopted by the working group
Chapter 7.4 ‘Leadless pacing’	*The following was added to the recommendations*: The 2016 NHRA guideline on implementation provides conditions for implanters and implanting centres, such as the minimum number of procedures and the presence of on-site cardiothoracic surgical back-up that need to be considered when following these recommendations
Chapter 8.2 ‘Pacing after cardiac surgery and heart transplantation’ (6) Patients requiring pacing after biological tricuspid valve replacement/tricuspid valve ring repair When ventricular pacing is indicated, transvenous implantation of a coronary sinus lead or minimally invasive placement of an epicardial ventricular lead should be considered and preferred over a transvenous transvalvular approach	*The following was added to the recommendation*: RV pacing is not necessarily the preferred option for permanent epicardial leads
Chapter 8.6 ‘Pacing in rare diseases’ In patients with cardiac sarcoidosis who have permanent or transient AVB, implantation of a device capable of cardiac pacing should be considered	*The recommendation was changed to*: In patients with cardiac sarcoidosis who have transient AVB, implantation of a device capable of cardiac pacing should be considered. In patients with cardiac sarcoidosis who have permanent AVB, implantation of a device capable of cardiac pacing is recommended
Chapter 11.1 ‘Performing magnetic resonance imaging in pacemaker patients’ In patients with MRI-conditional pacemaker systems, MRI can be performed safely following the manufacturer’s instructions. In patients with non-MRI-conditional pacemaker systems, MRI should be considered if no alternative imaging mode is available and if no epicardial leads, abandoned or damaged leads, or lead adaptors/extenders are present. MRI may be considered in pacemaker patients with abandoned transvenous leads if no alternative imaging modality is available	The recommendations from this chapter will not be adopted. We refer to the Dutch guideline module ‘*MRI bij elektronisch cardiaal implantaat*’ on this subject (authorised by NVVC in 2024)

*AF* atrial fibrillation, *AVJ* atrioventricular junction, *CRT* cardiac resynchronisation therapy, *HFpEF* heart failure with preserved ejection fraction, *AV* atrioventricular, *ICD* implantable cardioverter defibrillator, *NICM* non-ischaemic cardiomyopathy, *CSP* conduction system pacing, *RV* right ventricular, *HBP* His bundle pacing, *LBBAP* left bundle branch area pacing, *EHRA* European Heart Rhythm Association, *NHRA* Netherlands Heart Rhythm Association, *AVB* atrioventricular block, *MRI* magnetic resonance imaging, *NVVC* Dutch Society of Cardiology

### CRT in patients with persistent or permanent atrial fibrillation

The suggestion that CRT may be considered in patients with heart failure with preserved ejection fraction (HFpEF) from Chapter 6.3 ‘Cardiac therapy in patients with persistent or permanent atrial fibrillation’ has been clarified by the addition of: ‘Placing a CRT device has complications (the risk of complications is higher than with a 1-chamber or 2‑chamber device). It is important to make the decision regarding the installation of a CRT device through shared decision making.’ It has been demonstrated that the complication rate is higher in CRT than conventional pacing [3] and battery life is substantially shorter [4]. This needs to be balanced with the potential benefit of CRT and discussed with the patient. No prospective studies are available on CRT or CSP in patients with HFpEF. It has, however, been demonstrated in a subgroup analysis from the prospective PROSPECT trial that patients with an EF > 35% might benefit from CRT [5]. In addition, the APAF-CRT trial included patients with severely symptomatic permanent atrial fibrillation > 6 months, narrow QRS (≤ 110 ms) and at least one heart failure (HF) hospitalisation in the previous year [6]. Atrioventricular junction (AVJ) ablation plus CRT was superior to drug therapy in reducing both all-cause mortality and the secondary combined endpoint of death of any cause or HF hospitalisation. A benefit in all-cause mortality was observed in patients with preserved EF with no interaction between patients with EF > 35% and those ≤ 35%. Although this was not a comparison to right ventricular (RV) pacing, no previous trial or meta-analysis of AVJ ablation and RV pacing has shown a mortality benefit. Based on the findings of the APAF-CRT trial, it seems that patients fulfilling the inclusion criteria of the APAF-CRT trial should be considered for CRT irrespective of their LVEF.

### Upgrade from RV pacing to CRT

Regarding the recommendation from Chapter 6.4 ‘Upgrade from right ventricular pacing to cardiac resynchronisation therapy’, the working group would like to emphasise that the recommendation has now been confirmed by the results from the Budapest CRT trial [7]. This is the first RCT demonstrating the reduction of hard endpoints such as mortality, HF hospitalisation and reverse remodelling by upgrading from a conventional implantable cardioverter defibrillator (ICD) to CRT‑D in patients with significant RV pacing. The recommendation has not been adjusted.

### Benefit of adding an ICD in patients with an indication for CRT

A separate Dutch indication guideline has been published for primary prevention during ICD placement in patients with non-ischaemic cardiomyopathy. A reference to this guideline has been added to the recommendation from Chapter 6.6. The rationale of this guideline has been described in detail previously [8]. In summary, there is little evidence in favour of adding defibrillator therapy to CRT in patients with a non-ischaemic aetiology of heart failure. This has also been demonstrated in a recent meta-analysis [9].

### Conduction system pacing

The following two recommendations have been added to Chapter 7.2 ‘His bundle pacing’:

1. A proposed treatment with CSP versus conventional RV or CRT pacing should be discussed with the patient, weighing the advantages and disadvantages, including potential other short- and long-term complications (see Table 5 ‘Advantages and limitations of HBP and of LBBAP’ [EHRA clinical consensus statement on conduction system pacing implantation: endorsed by the Asia Pacific Heart Rhythm Society (APHRS), Canadian Heart Rhythm Society (CHRS), and Latin American Heart Rhythm Society (LAHRS) | Oxford Academic (oup.com)] from the EHRA consensus document [10]).
2. The recommendations on the practical application of CSP as stated in the Table of advice chapter from the EHRA Consensus document [10] have been adopted by the working group.

The current ESC guideline ‘Cardiac pacing and cardiac resynchronisation therapy’ [1] makes limited recommendations on His bundle pacing (HBP) and no recommendations are given on left bundle branch area pacing (LBBAP) given the lack of sufficient data on this modality at the time. However, since 2021, numerous predominantly observational studies on these forms of CSP have been published. In 2023, (1) a European Heart Rhythm Association (EHRA) clinical consensus statement on CSP implantation was published [10] and (2) a document was drawn up by non-European international heart rhythm societies with recommendations on CSP based on this recent literature [11]. An EHRA expert consensus document on when to use CSP is expected in 2025, providing more guidance on the use of this emerging technique.

In the Netherlands, CSP (and LBBAP in particular) has been increasingly applied in clinical practice in recent years, despite the lack of results from ongoing large RCTs. The field therefore needs advice on applying LBBAP pending these results (and an update in the ESC guideline). Given the planning published on the ESC website, an update of the current 2021 guideline is not expected before 2027.

Taken together, the working group has decided to extend the current recommendations from the ESC guideline (where possible) for Dutch practice. The working group wanted to provide scope for applying LBBAP. Consideration has been given to conducting a literature analysis. However, current evidence comes mainly from observational studies. In order to be able to draw up recommendations about LBBAP with any certainty, the results from ongoing RCTs are needed.

### Leadless pacing

The recommendations from Chapter 7.4 have been endorsed by the working group. It should be noted that the Netherlands Heart Rhythm Association published an i implementation guideline in 2016 that provides conditions for implanters and the implanting centre under which this technique may be safely used [*Intracardiale pacemaker* (www.nvvc.nl)]. It formulates recommendations on training and the minimum number of procedures per operator and centre but also recommends the presence of on-site cardiothoracic surgical backup.

### Pacing after cardiac surgery and heart transplantation

The recommendation from Chapter 8.2 ‘Pacing after cardiac surgery and heart transplantation’ has been clarified by the addition of: RV pacing is not necessarily the preferred option for permanent epicardial leads.

Epicardial pacing leads have been used extensively for CRT; they have good longevity and should be placed in the posterolateral region [12]. In addition, it has been shown that pacing from an epicardial position can be performed better from the LV apex in children. Given the better synchronicity it can be hypothesised that this is also true for adults [13].

### Pacing in rare diseases (Chapter 8.6)

The indication for a device capable of cardiac pacing for permanent atrioventricular block (AVB) is evident in patients with cardiac sarcoidosis in analogy to patients with a different aetiology of AVB. Therefore, the recommendation from Chapter 8.6.6.1 on cardiac sarcoidosis has been amended to recommend implantation of a device for permanent AVB in patients with cardiac sarcoidosis instead of ‘should be considered’.

### Performing magnetic resonance imaging in patients with a pacemaker or ICD

**Fig. 1:**
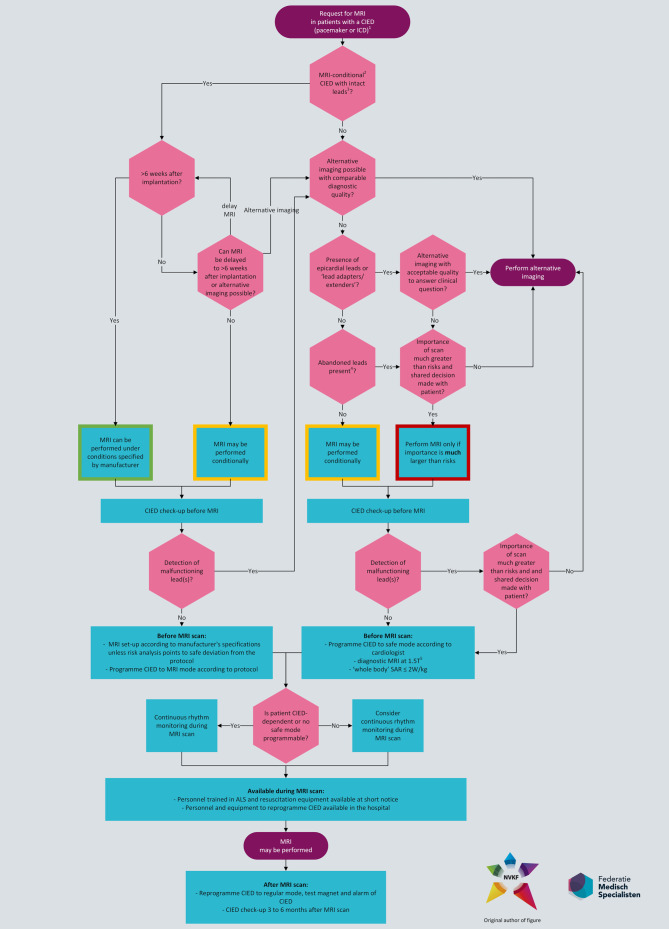
Magnetic resonance imaging in patients with cardiac implantable electronic devices according to therecommendations of the Dutch guideline module ‘*MRI bij elektronisch cardiaal implantaat*’ (https://richtlijnendatabase.nl/richtlijn/gebruik_mri_bij_patienten_met_implantaten/mri_bij_elektronisch_cardiaal_implantaat_2024.html), copied from the guideline ‘*Gebruik van MRI bij patienten met implantaten*’ with permission from the Dutch Association of Clinical Physicists. ^1^External pacemakers are not covered by this guideline; see the Considerations section; ^2^There are no MRI-safe CIEDs, only MRI-conditional or non-authorised ones; ^3^There are no epicardial leads, without extenders or lead adapters; ^4^Abandoned leads include capped or cut leads and lead fragments; ^5^‘Whole body’ MRI system with horizontal/closed superconducting magnet with a field strength of 1.5 T. *ALS* advanced life support, *CIED* cardiac implantable electronic device, *ICD* implantable cardioverter defibrillator, *MRI* magnetic resonance imaging, *SAR* specific absorption rate, *T* Tesla

For recommendations on performing magnetic resonance imaging (MRI) in patients with a pacemaker or ICD, we refer readers to the Dutch guideline module on this subject (authorised by the NVVC in 2024): https://richtlijnendatabase.nl/richtlijn/gebruik_mri_bij_patienten_met_implantaten/mri_bij_elektronisch_cardiaal_implantaat_2024.html. This guideline incorporates more recent literature and gives more detailed practical advice than the current ESC guidelines. There seem to be few contraindications for MRI if the correct precautions are taken. Even patients with abandoned leads or epicardial leads are at low risk when undergoing MRI [14]. The workflow for patients with a pacemaker or ICD where an MRI is requested is summarised in Fig. 1.

The recommendations from Chapter 11.1 will therefore not be applicable for Dutch practice.

## Summary of patient perspective

To gather the perspectives of patients with a pacemaker (PM) or CRT device, Harteraad conducted a literature search, held in-depth interviews with six patients and analysed contributions in a closed community for patients with cardiac arrhythmia. Findings from all three sources have been combined in this summary. For more details, we refer to the published addendum.

The complaints and limitations experienced by patients due to cardiac arrhythmia differed substantially. Some patients could not work anymore, while others were limited to some extent by the condition. However, all patients hoped that a device would have a positive effect on these complaints and limitations.

After placement of a device, patients had different experiences regarding the advantages and disadvantages. Some patients were back to their old self, others did not see a change. Some patients experienced fear, which could be for different reasons. However, the majority of patients indicated that the benefits outweighed the side effects.

Patients generally experienced a lack of SDM in determining the potential treatment options. Many patients stated that there was no conversation on (alternative) treatment options, including their benefits and harms. They received no information about the procedure and therefore did not know what to expect. They were also not informed about recovery, aftercare and rehabilitation. They also found the explanation they received from their doctor rather difficult to understand. In retrospect the patients expressed trust in their doctor for making a deliberate decision that a PM/CRT device was needed for their situation. Combining information from the above stated sources, patients expressed their need for understandable information about:

- their condition, test results and treatment options, including the pros and cons.
- what treatment with a PM/CRT device entails, what effect, risks and implications for daily life they can expect.
- the course of events surrounding the implantation of a PM/CRT device.
- recovery, aftercare and rehabilitation.
- practical tips for daily life with a heart rhythm disorder and a PM/CRT device.
- advice on lifestyle adjustments.

The patients that participated in the survey were rather young compared to the general population receiving pacemakers. This should be taken into account when interpreting their perspective. It would be important to perform further research into the perception of the decision-making process and how it could be improved.

## Patient-centred care and SDM in cardiac pacing and CRT

The working group would like to emphasise the recommendation from Chapter 12: In patients considered for a PM or CRT device, the decision should be based on the best available evidence with consideration of individual risk/benefits of each option, the patient’s preferences and goals of care. It is recommended that an integrated care approach be followed and that the principles of patient-centred care and SDM be used in the consultation. For this addendum, a qualitative interview study was conducted among future and former cardiac device patients ≥ 18 years (see Summary of patient perspective). This study emphatically showed the patients’ need for SDM. However, SDM is not yet applied sufficiently within this group, specifically in acute situations. Futhermore, the study indicates what kind of information a patient needs, emphasising specifically information about the extent to which cardiac devices influence the patients’ daily life. It is therefore essential that health professionals have insight into a patient’s own living situation; this should thus be discussed for SDM to be successful. It should be noted that the patients interviewed partly overlap with the population from the ESC guideline.

The results of the study are in line with the definition of SDM. SDM is a joint process in which a healthcare professional works together with the patient to a reach a decision about care. It involves choosing tests and treatments based both on evidence and on the person’s individual preferences, beliefs and values. It is important that social and psychological environments as well as the functional status of patients are taken into account in the decision-making process.

The NVVC has established a working group to prioritise further development of the implementation of SDM in the workplace.

## Summary and conclusions

Most of the current ESC guidelines on cardiac pacing and CRT can be endorsed with some amendments as well as omissions and additions. For ICD therapy in non-ischaemic cardiomyopathy, the new Dutch practice guidelines should be followed. Patients with a pacemaker or ICD that have to undergo MRI should be advised in accordance with the new Dutch guideline which is more extensive than the ESC guideline. SDM is of utmost importance and physicians should implement diagnostics and therapy on the basis of evidence but also incorporating the patients’s individual preferences, beliefs and values.
